# Chinese electricity-focused input-output dataset with detailed coal power and alternative energy for 2018

**DOI:** 10.1038/s41597-023-02466-8

**Published:** 2023-08-22

**Authors:** Yunsong Liang, Yuning Zhang, Yanhua Wang, Hongxia Zhang, Ke Wang, Zhanming Chen

**Affiliations:** 1 School of Applied Economics, Renmin University of China, Beijing, 100872 China; 2 School of Environment and Natural Resources, Renmin University of China, Beijing, 100872 China

**Keywords:** Environmental economics, Socioeconomic scenarios

## Abstract

The electricity-focused input-output model is a popular approach for analysing the socio-economic and environmental impacts of electricity decarbonisation policies; however, it cannot be built directly owing to a lack of data on electricity technology. Here, we provide the Chinese electricity-focused input-output dataset, which characterises the production and distribution of 14 electricity subsectors. Based on the official input-output table for China in 2018, we disaggregate the original electricity sector by referring to macro data from statistics departments and our micro data on the unit-level cost information of China’s coal power. This is China’s most recent electricity-focused input-output dataset, featuring novel improvements in sub-electricity identification, especially mapping six detailed coal power sources and six alternative power sources. The Chinese electricity-focused input-output dataset can be used as the baseline for extensive satellite account compilation, allowing for a variety of in-depth studies on footprint analysis and policy simulations related to China’s electricity transition.

## Background & Summary

As the dominant electricity producer globally, China has rapidly changed its energy mix for electricity production over the past decades. From 2012 to 2022, the percentage of coal generation decreased from 75.5% to 61.3%, while the generation share of wind and solar power increased from 2.1% to 13.9%^1^. Nevertheless, the electricity and steam sector was responsible for 5789 Mt of CO_2_ emissions in 2021^2^, accounting for nearly half of the total emissions from national fossil fuel combustion. In the process of phasing down coal power, it is imperative to categorise coal-fired power plants of varying sizes^3^, as they assume distinct roles in terms of electricity generation and emissions reduction^4^. A low-carbon electricity transition will strongly support China’s short- and mid-term climate mitigation strategy, ‘Action Plan for Carbon Dioxide Peaking Before 2030’, with a focus on new coal power restrictions and massive renewable source substitution^5^. Deep decarbonisation of the electricity sector would be far-reaching in achieving China’s climate targets^6,7^, energy efficiency^8,9^, environmental benefit^10,11^, and socio-economic welfare^12,13^. To analyse the energy-environment-economy impacts of electricity transition, the electricity-focused input-output (EFIO) model is one of the most used top-down methods for tracing industrial linkage effects^14–17^.

The foundation of the EFIO model is an economic input-output table (IOT) with heterogeneous electricity subsectors, including electricity supply and production from combustible fuels (coal, natural gas, etc.) and renewable sources (wind, solar, hydroelectricity, etc.). Coal power must be distinguished in an IOT, particularly in China, where it is the main source of electricity production and has significant heterogeneity in generation efficiency. However, official Chinese IOTs involve only one aggregate electricity sector. The loss of detailed information about the production mix of subsectors leads to an ‘aggregation bias’^18–20^. A commonly effective solution is to integrate bottom-up technological approaches (such as Life Cycle Assessment) with rectangular supply and use tables (SUTs). Rather than directly disaggregating the original IOT, this approach accurately tracks the production and consumption of multiple electricity commodities and contributes to the creation of a new IOT^21–23^. However, China’s use table is compiled based on the supply table and the IOT due to various limitations^24^. Consequently, Chinese official IOTs are not solely derived from SUTs, making the disaggregation rules from the SUT framework less applicable. Therefore, alternative methodologies starting from symmetric IOTs are widely used and developed in the Chinese context. In this regard, researchers usually disaggregate the original electricity sector following Wolsky’s approach^25^ when the power generation and electricity prices of new subsectors are partially available^14,26,27^. Lindner *et al*. further extended Wolsky’s approach by referring to the operational cost of power plants and regional information of the grid system to compile the Chinese EFIO table for 2007^28,29^, and Li *et al*. adopted a similar approach to construct Chinese EFIO tables for 2012 and 2017^8^. However, there are several limitations to the previous disaggregation process that has been in use. First, these studies focused on disaggregating renewable power from electricity but failed to delve into the discrepancy between coal-fired electricity at the unit level. Second, limited data on the operation and maintenance costs of power guided the clarification of new interindustry transactions. Third, some studies ignored statistical calibres and standards and relied heavily on mathematical algorithms, which may have not been realistic. Moreover, these developed EFIO tables were compiled mostly for the year before 2017 and, thus, cannot clarify China’s new electricity mix.

To better understand the relationship between China’s electricity and economic sectors, we built the Chinese electricity-focused input-output (CEFIO) dataset for 2018 based on the official IOT^30^. In the CEFIO dataset, we disaggregated coal power from electricity as closely as possible by referring to the previously collected unit-level cost information of China’s coal power^31^. To trace the technical characteristics of transmission and distribution (T&D) and renewable power, we used macro data from official governments and industrial associations in China on the electricity mix (such as abandonment rate) and business activities (such as sales revenue)^32–35^. Following our previous work^36^, we focused on complying with the accounting principles of the input-output sectors during the disaggregation process. Therefore, the electricity sectors in the CEFIO dataset comprise 14 detailed sectors: a T&D sector, a steam sector, and 12 electricity production sectors (six different coal-fired powers at the unit level, three typical renewable powers, and nuclear power, other thermal power, and other electricity production).

To the best of our knowledge, the CEFIO dataset that we developed for 2018 is the only up-to-date EFIO dataset and covers the most diverse electricity subsectors in China. This is also the only publicly available EFIO dataset that can be freely downloaded from an online platform (10.6084/m9.figshare.c.6119313)^37^. We provide this in 166- and 55-sector classifications, with economic transactions characterised in Chinese yuan (CNY). The dataset can support, including but not limited to, the following research: (1) identifying electricity classification for compiling satellite accounts such as carbon emissions, water consumption, and employment; (2) quantifying the environmental footprints of China’s electricity subsectors by building extended environmental input-output models; and (3) evaluating the policy impacts of the coal-fired power phase down and renewable energy transition in China to achieve carbon dioxide peaking and carbon neutrality targets.

## Methods

This section describes the disaggregation structure, principles, data sources, procedures, and steps involved in constructing the CEFIO dataset.

### Disaggregation structure

The objective of this study was to disaggregate the *Production and Supply of Electricity and Steam* sector into 14 new sectors (Table 1). The original IOT to be disaggregated was a high-resolution IOT with 153 sectors. Thus, the CEFIO dataset was further disaggregated into 166 sectors. The original IOT cannot be used to effectively analyse the transformation of the electricity structure for carbon peaking and carbon neutrality goals because of two shortcomings: (1) it fails to distinguish various types of power generation; and (2) two parts of the original sector, *Power Transmission and Distribution* and *Production and Distribution of Steam*, do not belong to power generation, which makes the analysis more difficult. To overcome these shortcomings, the new sectors in the CEFIO dataset can be classified into three categories: (1) electricity production, (2) T&D, and (3) steam. The second and third categories each contain one new sector. In electricity production, the initial distinction is made based on different power generation technologies. This differentiation is crucial because varying technologies result in substantial differences in demand for upstream sectors. Moreover, the subdivision of coal-fired power into different installed capacities considers two key factors: the prominent share of coal-fired power within China’s power sector and the significant heterogeneity in energy efficiency among coal-fired power plants with varying installed capacities. Notably, cogeneration, which is a common occurrence in China, is a part of thermal power and not a separate sector in the CEFIO dataset. This method is the same as the ones in nearly all of the previous studies. Consequently, electricity production consists of 12 new sectors: six kinds of coal-fired units with different installed capacities, other thermal power, hydropower, nuclear power, wind power, solar power, and other electricity production. Referring to the statistical classification of the China Electricity Council (CEC), coal power is classified into six categories: <100 MW, 100–200 MW, 200–300 MW, 300–600 MW, 600–1000 MW, and ≥1000 MW.

**Table 1:** List of sectors related to disaggregation steps.

Original sector	Second-level sectors	Third-level sectors	New sectors
Production and Supply of Electricity and Steam	Thermal Power	Coal Power	Coal Power (<100 MW)
			Coal Power (100–200 MW)
			Coal Power (200–300 MW)
			Coal Power (300–600 MW)
			Coal Power (600–1000 MW)
			Coal Power (≥1000 MW)
		Other Thermal Power	Other Thermal Power
	Hydropower	Hydropower	Hydropower
	Nuclear Power	Nuclear Power	Nuclear Power
	Wind Power	Wind Power	Wind Power
	Solar Power	Solar Power	Solar Power
	Other Electricity Production	Other Electricity Production	Other Electricity Production
	Power Transmission and Distribution	Power Transmission and Distribution	Power Transmission and Distribution
	Production and Distribution of Steam	Production and Distribution of Steam	Production and Distribution of Steam

### Principles

To depict the heterogeneity in power generation and improve data quality, three principles were proposed and followed during CEFIO dataset construction. First, we prioritised standards. This was reflected in two ways. In terms of the sector setting, we drew lessons from the Industrial Classification for National Economic Activities and the explanation for the sector classification of China’s 2018 IOT compiled by the Department of National Accounts (DNA) of the National Bureau of Statistics (NBS)^30,38^. For raw data selection, the cited indicators were determined based on the latest compilation method used by the NBS^24^. Since the original IOT was also compiled by the NBS, this principle ensured the scientific nature of our method and the consistency of our dataset. Second, we considered constraints. The term ‘constraints’ refers to the gross output and the value-added at the sector level, which imposes constraints on the IOT during balancing. Focusing on constraints, we prioritised and estimated the gross output and value-added of the new sectors using various available raw data and then checked the rationality in time to ensure the accuracy of the results. Once these aggregate indicators were accurately estimated, the estimation of the intermediate flows was controlled, which means that the bias would not be too large. Third, we combined the micro- and macro-level data. This is valuable for unofficial methods of compiling IOTs. The use of micro data of coal power firms^31^ not only depicts this sector’s heterogeneity, but also checks the macro data used to improve data quality.

### Data collection

We used three raw data sources. The first was macro data from the statistics department, including China’s 2018 IOT, the Fourth National Economic Census, and the *China Industry Statistical Yearbook*^30,32,33^. The IOT was a benchmark, whereas the latter two provided an important reference for preliminary disaggregation. The second was the statistics from the CEC^34,35^. CEC data are usually inconsistent with those from the statistics department. Considering the advantages of the two data sources, macro data are preferred in this method, and data from the CEC are used for disaggregation when the macro data cannot provide more detailed structural information. The third was the unit-level cost database for China’s coal power that we previously constructed^31^. This database integrates data on the operational status, financial cost information, and profitability of coal power projects at different scales, technologies, and regions. It provides abundant micro data for dataset construction, such as the cost structure of different unit levels of coal power plants and house-service consumption. This significantly enhanced the resolution of the coal power sector in the CEFIO dataset.

Notably, the macro data sources are provided by the industry, while China’s official IOT is a commodity-by-commodity table. In contrast to the ‘industry’ sector, the ‘commodity’ sector in the input-output model (or in an IOT) means that the sector can only produce one commodity. Typically, the process of transforming raw data from industry sectors to commodity sectors is accomplished by utilising the supply table. However, a disaggregated supply table that can identify sub-electricity is not available. We could not refer to other data to complete the transformation. Therefore, to use the data sources to complete the disaggregation, we had to assume that the disaggregated sectors are ‘pure’ sectors, each producing only one commodity. The raw data could then be introduced directly.

### Outline of procedures

**Fig. 1:**
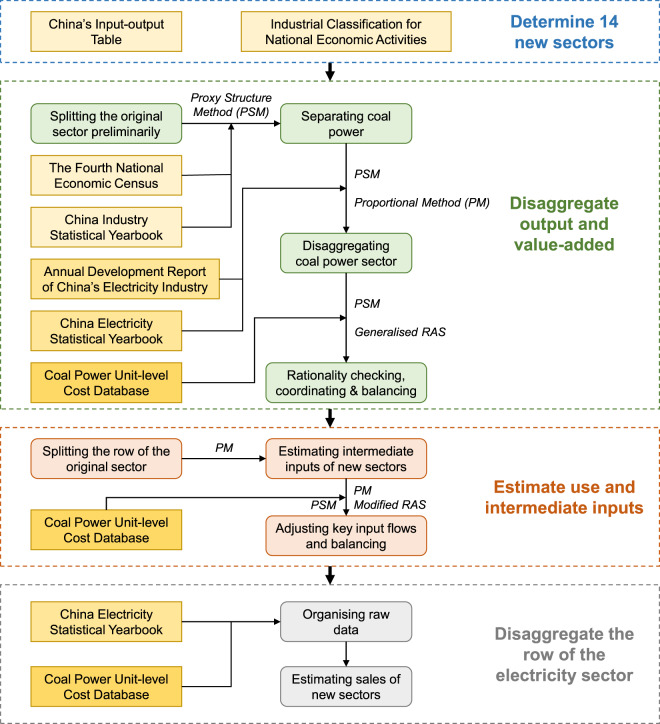
Flowchart of the Chinese electricity-focused input-output dataset construction. The Generalised RAS and Modified RAS are methods for input-output table balancing.

Generally, there are four steps in establishing the CEFIO dataset: (1) determining the new classification, (2) disaggregating output and value-added data, (3) estimating use and intermediate inputs, and (4) further disaggregating the row of the electricity sector. Figure 1 shows the entire process of the construction of the CEFIO dataset.

First, we developed a new classification system. According to the relevant standards and data conditions, we determined 14 new sectors that were subdivided from the original sector, including 12 electricity production sectors, one T&D sector, and one steam sector.

Second, output and value-added were disaggregated. Estimating the constraints of new sectors, specifically gross output and value-added, lays the foundation for subsequent disaggregation. Accurately estimating the constraints allows for improved clarity and lower error risk. Considering the availability of raw data, this step was further divided into four steps: (1) disaggregating the gross output and value-added of the original sector into eight second-level sectors; (2) separating coal power from thermal power to obtain nine third-level sectors; (3) further disaggregating the coal power sector into six types of units to obtain 14 new sectors; and (4) rationality checking, coordinating, and balancing. Table 1 provides a breakdown of the original sector into new sectors.

Third, we estimated use and intermediate inputs. In this step, we estimated the sales destinations (row information) and intermediate inputs (column information) of the new sectors, with the sales destinations of the electricity sectors (including production and T&D) as homogeneous. In other words, from the perspective of the row, we treated these sectors related to electricity as one and called it ‘the electricity sector’. Therefore, the row of the original sector was divided into two, and its column was completely disaggregated. Specifically, there were three steps: (1) splitting the row of the original sector into two rows according to a given proportion, (2) dividing the column of the original sector into 14 columns according to another given proportion, and (3) adjusting the key input flows and balancing.

Fourth, the row of the electricity sector was further disaggregated. Using our micro data and summarising the features and statistical rules of power systems in China, we estimated the intermediate sales of each new sector; that is, new sector goods as intermediate inputs used by all sectors. There were two steps: (1) organising raw data and (2) estimating the sales of new sectors according to electricity features.

**Fig. 2:**
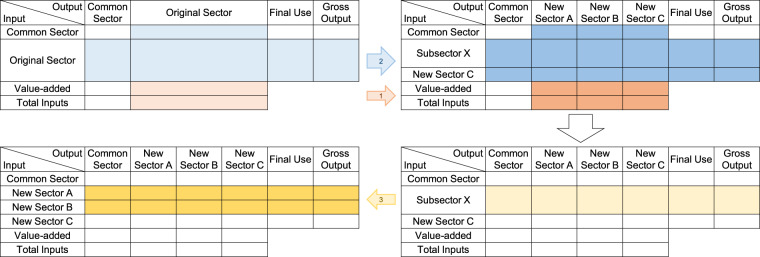
Schematic diagram of the disaggregation process. In this figure, the original sector was divided into three new sectors. The new sectors, A, B, and C are electricity production, T&D, and steam, respectively. Subsector X is the electricity sector mentioned in the text.

After completing these steps, we obtained the CEFIO dataset with 166 sectors. Figure 2 shows a schematic of the disaggregation from the viewpoint of the block structure of the IOT.

### Disaggregating output and value-added

The CEFIO dataset is essentially a further processing of the original IOT, and there is no need to recalculate gross output and value-added. Therefore, it is logical to select appropriate proxies for indicators and disaggregate the original sector based on the proportional relationship between the comparability data of each subsector’s proxy and their summation. The formula is expressed in Eq. 1:

1 $$\begin{array}{c}{x}_{j}={w}_{j}X=\frac{{p}_{j}}{{\sum }_{j}{p}_{j}}X=\frac{{p}_{j}}{P}X\end{array}$$

where *x*_*j*_ and *X* refer to the indicators (such as output, value-added, intermediate inputs, etc.) of the lower-level sector *j* and the sector to be divided, respectively, for example, a second-level sector and the original sector, or a type of unit and coal power; *w*_*j*_ refers to the weight of lower-level sector *j* in its higher-level sector; and *p*_*j*_ and *P* refer to the proxies of the two sectors above. The term ‘proxy’ refers to the statistical indicators that have two characteristics: (1) data availability, and (2) consistency of meanings compared with the indicator to be processed. This method, which uses Eq. 1, was named the Proxy Structure Method (PSM). The selection of proxies is at the core of the PSM. The consistency of meanings is extremely critical and directly relates to the scientific nature of the method and the rationality of the results. However, the data availability imposes certain constraints. In other words, there is a trade-off when selecting each proxy. The PSM was mainly applied to disaggregate the output and value-added data (Fig. 1).

#### Splitting the original sector preliminarily

Referring to the official compilation method^24^, we extracted the accounting principles of gross output, value-added, and other indicators and selected proxies according to these principles. Some financial indicators were chosen as proxies. Considering the data available, we used data from the Fourth National Economic Census and the *China Industry Statistical Yearbook*^32,33^. Table 2 lists the indicators and proxies used in this step.

**Table 2:** List of indicators and their proxies involved in the disaggregation of the original sector.

Indicators	Proxies
Gross Output	Sales Revenue
Value-added (The sum of the four items below)	None
Compensation of Employees	Average Number of Employees
Net Taxes on Production	Sales Revenue - Cost of Sales - Expenses + Investment Income - Operating Profit
Depreciation of Fixed Assets	Accumulated Depreciation in the Current Year - Accumulated Depreciation in the Previous Year
Operating Surplus	Operating Profit

The proxies were selected based on the Fourth National Economic Census. Only the ‘Accumulated Depreciation in the Previous Year’ is from the *China Industry Statistical Yearbook*.

#### Separating coal power from thermal power

This step divided thermal power (a second-level sector) into coal power and other thermal power (two third-level sectors) in terms of gross output and value-added. Due to data limitations, we could only use the same proportion to disaggregate gross output and value-added (including its components). Specifically, proxy selection was based on gross output, and the weight *w*_*j*_ was calculated using Eq. 1 and applied to disaggregate all the indicators in this step. The proxy chosen was the product of the amount of electricity generated through a certain technology (i.e. coal power or other thermal power) and its price. The amount and price of electricity were obtained from the *China Electricity Statistical Yearbook* and *Annual Development Report of China’s Electricity Industry*, respectively^34,35^.

#### Disaggregating coal power sector

This step further disaggregated the gross output and value-added of coal power (a third-level sector) into six units (new sectors). Based on our unit-level cost database^31^, we selected the proxies required in this step, as listed in Table 3. Notably, the proxy for gross output included heat sales revenue, which was consistent with our treatment of cogeneration.

**Table 3:** List of indicators and their proxies involved in the disaggregation of the coal power sector.

Indicators	Proxies
Gross Output	Electricity & Heat Sales Revenue
Value-added (The sum of the four items below)	None
Compensation of Employees	Wage of Employees
Net Taxes on Production	Value-added Tax + City Maintenance and Construction Tax & Education Surcharge - Input Tax Deduction of Fixed Assets
Depreciation of Fixed Assets	Depreciation Charge
Operating Surplus	Profit before Tax

The proxies were selected based on the unit-level cost database. Though the proxies are not the same as those in Table 2, the principle of proxy selection remained unchanged.

#### Rationality checking, coordinating, and balancing

Significantly, we did not consider whether the disaggregation results were rational within the new sectors. In other words, disaggregation occurred in the horizontal direction in the IOT, whereas the results were not examined in the vertical direction. Once the column structure is considered, information conflicts are likely to occur because of the multi-source data. Therefore, a rationality check and manual coordination are necessary. Specifically, the irrational structure of the new sector’s value is adjusted, and the matrix of value-added as a whole is balanced. Thus, the structural information provided by the raw data can be retained as much as possible, and errors can be reduced. Considering that there may be negative entries in the value-added component, we used the Generalised RAS (GRAS) algorithm to balance the matrix^39,40^.

Taking the first-step results as an example, Table S1 presents the value-added (including four components), total inputs, and value-added rates of the eight second-level sectors before and after rationality checks. Based on expert judgement, the value-added rate of the steam sector seemed too low, mainly due to the large negative value of its net taxes on production. Thus, we adjusted it and rebalanced the value-added matrix of the second-level sectors, keeping their gross output (i.e. total inputs) unchanged. The result after checks showed that the value-added (with its components) of other second-level sectors was not significantly affected, and the overall structure of the PSM result was preserved.

### Estimating use and intermediate inputs

After disaggregating the constraints (gross output and value-added), we focused on the sales destinations and intermediate inputs of the new sectors. Because there were many flows to be estimated and limited raw data that could be referred to, the Proportional Method (PM) was used in this step, which involved splitting a column or row with a given proportion. The PM will not change the balance of the IOT and cannot introduce new information. It is a common simplified method for disaggregating IOTs that assumes that the split rows or columns have the same proportional relationship. In other words, the structure obtained through splitting is homogeneous. However, a homogeneous structure, particularly a homogeneous production structure, is undesirable. Thus, in addition to the PM, we used the PSM and checked the rationality again to adjust the key input flows by introducing micro data, and then balanced the matrix of intermediate flows using the Modified RAS (MRAS)^41^. In this step, the original sector was divided into 14 columns, but its row was divided into two: the electricity and steam sectors. The result of this step was a semi-finished product because an input-output model requires the technical coefficient matrix to be a square.

#### Splitting the row of the original sector

The row of the original sector was split; that is, the sales destinations of the electricity and steam sectors were estimated. Without additional data support, it was assumed that the distribution structures of these two sectors were the same and consistent with those of the original sector. The formula used in this step is expressed in Eqs. 2–3:

2 $$\begin{array}{c}{z}_{ij}={w}_{ij}{x}_{i}=\frac{{z}_{Ij}}{{x}_{I}}{x}_{i},\,\forall j\end{array}$$

3 $$\begin{array}{c}{f}_{ik}={w}_{ik}{x}_{i}=\frac{{f}_{Ik}}{{x}_{I}}{x}_{i},\,\forall j\end{array}$$

where *z* and *f* refer to intermediate and final use, respectively; *x* refers to gross output; *I* refers to the row of the original sector; *i* refers to the row of the disaggregated sector (i.e. the electricity sector or the steam sector); *j* refers to the column of any sector; *k* refers to the type of final use (e.g. rural household consumption expenditure); *z*_*ij*_ represents interindustry sales by sector *i* to sector *j*; and *f*_*ik*_ represents sales by sector *i* to type *k* of final use. This step is equivalent to splitting all flows of the original sector row according to the shares of the two disaggregated sectors’ outputs, which also means that the structures of the two sectors in each column (including the intermediate inputs of each sector and each category of final use) are the same. The proportionality assumption was acceptable for three reasons: (1) the output of the steam sector accounted for less than 4% of that of the original sector; (2) steam is widely used as the main source of indoor heating by various sectors in China; and (3) there was not enough information describing steam’s distribution.

#### Estimating intermediate inputs of new sectors

In this step, the production structure of the new sector was estimated. It was unrealistic to split and adjust each sector because there were numerous entries in the matrix of intermediate inputs. Thus, the PM was used to provide the initial estimation. Since the gross output and value-added of each new sector had been determined, the PM assumed that the intermediate input structures (i.e. the proportions of each intermediate input in the total intermediate inputs) were the same among new sectors and were also consistent with the original sector. Equation 4 shows the formula used:

4 $$\begin{array}{c}{z}_{ij}={s}_{ij}({x}_{j}-{v}_{j})=\frac{{z}_{iJ}}{{x}_{J}-{v}_{J}}({x}_{j}-{v}_{j}),\forall i\end{array}$$

where *z* and *v* refer to intermediate inputs and value-added, respectively; *J* refers to the column of the original sector; *j* refers to the column of any new sector; *i* refers to the row of any sector; and *z*_*ij*_ represents intermediate inputs purchased by sector *j* from sector *i* (which is the same as interindustry sales by sector *i* to sector *j*). This is equal to disaggregating all flows in the original sector’s column based on the shares of total intermediate inputs of new sectors, implying that the sales structures from each sector to the new sectors are identical. The results required further adjustments.

#### Adjusting key input flows and balancing

Considering the sizes of the technical coefficients and heterogeneity of the inputs for various types of electricity production, we selected 11 key sectors for manual adjustment. These sectors were classified into four categories: (1) fuel and auxiliary materials, (2) equipment, (3) services, and (4) special cases (including electricity transactions and losses).

Directly relying on raw data is difficult for two reasons. First, given the concept of technical coefficients, it is difficult to find corresponding data for reference. Second, the information provided by the data from different sources may conflict, making the results of the adjustment from the horizontal and vertical directions inconsistent. Therefore, we combined the PSM, rationality checks, and adjustments when introducing the raw data. Table 4 shows the details of the adjustment. As can be observed, we developed adjustment principles based on the background of the new sectors’ production, such as the fact that electricity production does not use coal except for coal power, T&D does not use boilers, and so on. Steam is produced in China by using coal, oil, gas, and other energy sources via boilers and other devices^30,38^. Therefore, we adjusted its input structure to resemble that of thermal power generation.

**Table 4:** List of adjustment principles of key flows. Key flows refer to the sales by 11 key sectors to 14 new sectors, which are also the intermediate inputs of the new sectors.

Categories	Key sectors (rows)	Adjustment principles
Fuel and Auxiliary Materials	Mining and Washing Products of Coal	Only in *Coal Power* and *Steam*; referred to our micro data^31^
	Extraction Products of Crude Petroleum and Natural Gas	Only in *Other Thermal Power* and *Steam*
	Refined Petroleum Products, Nuclear Fuel Processing Products	Mainly in *Nuclear Power* (38%) and *Other Electricity Production* (29%)
	Coal Processing Products	Only in *Thermal Power* and *Steam*; mainly in *Other Thermal Power* (39%) and *Steam* (40%)
	Production and Distribution of Gas	Mainly in *Other Thermal Power* (17%) and *Steam* (28%)
	Production and Distribution of Water	Slightly larger in low-power units (*Coal Power*) (11%) and *Steam* (22%)
Equipment	Boiler and Prime Mover	None in *T&D*
	Equipment for Power Transmission and Distribution and Control	None in *Steam*; slightly larger in *T&D* (73%) and *Solar Power* (5%)
	Measuring Instruments	Slightly larger in *Renewable and Nuclear Power* (11%)
Services	Monetary Intermediation and Other Financial Services	Slightly larger in *Renewable and Nuclear Power* (7%); referred to our micro data^31^
Special Cases	Electricity	Mainly in *T&D* (sales by plants to the grid) (93%); referred to our micro data^31^

The percentages in the table refer to the shares distributed to a new sector in all new sectors’ purchases from the key sector during the adjustment. They are not necessarily the final result because of balancing. ‘Mainly’ and ‘slightly larger’ are relative to proportionality (i.e. the proportion of total intermediate inputs in new sectors to their total).

The key input flows were adjusted manually according to the principles and relevant raw data. This adjustment destroyed the balance of the IOT; therefore, balancing was required. We used the MRAS to exclude some entries from balancing. We kept seven sectors (rows) unchanged: *Mining and Washing Products of Coal*, *Extraction Products of Crude Petroleum and Natural Gas*, *Refined Petroleum Products*, *Nuclear Fuel Processing Products*, *Coal Processing Products*, *Production and Distribution of Gas*, *Monetary Intermediation and Other Financial Services*, and *Electricity*. These flows were selected because of their relatively clear economic implications and strong raw data support. We obtained an informative, balanced but non-square IOT with 154 sectors in the horizontal direction and 166 sectors in the vertical direction.

### Disaggregating the row of the electricity sector

As mentioned earlier, a non-square IOT is inconvenient to use. In this step, we further disaggregated the row of the electricity sector by employing micro data and capturing the features of electricity supply and use. The twin goals of this step were to: (1) obtain a square matrix of technical coefficients and (2) make the flows as economically meaningful and realistic as possible.

#### Organising raw data

The data required here included two aspects: the line loss rate (LLR) and the share of captive power plants (CPPs). The former describes the loss in the T&D process (i.e. the intraindustry input of the *Power Transmission and Distribution* sector). The latter distinguishes the different sources of electricity used by non-electricity sectors (i.e. from the grid or their CPPs). The LLR data were obtained from the *China Electricity Statistical Yearbook*^34^ and did not require processing. The CPP data were plant-unit-level data from our cost database of China’s coal power^31^, which needed organising. The core task was to identify CPPs by their names, match their corresponding sectors, and aggregate the data. Through processing and organising, we collected sector-unit-level data on CPPs. To maintain consistency with the IOT, we only took the structural information of the raw data and controlled the entries using the IOT.

#### Estimating sales of new sectors

This step divided the electricity sector row into 13 rows. To reflect the characteristics of electricity supply and use in the CEFIO dataset, we generalised the features and statistical rules of China’s power systems and disaggregated the row of the electricity sector based on them. We summarised the following five features:

- Feature 1: **Except for those with CPPs, non-electricity sectors** consume electricity from the grid.
- Feature 2: **Non-electricity sectors with CPPs** consume most of their electricity from the grid, with the remainder originating from their CPPs (mostly coal power).
- Feature 3: **Electricity production sectors** consume electricity from themselves rather than the grid (so-called house-service consumption).
- Feature 4: **The grid** purchases electricity from power plants and supplies it in a lossy way to all users (except power plants). The transaction is naturally recorded as revenue for power plants as well as the cost of the grid, which in turn becomes a part of its gross output (total inputs).
- Feature 5: **All final use** of electricity comes from the grid.

Based on these features, we estimated the sales of the new sectors in the CEFIO dataset. This step maintained the balance of the IOT, and therefore balancing was not required. Further details are presented in Table 5.

**Table 5:** Steps of disaggregating the electricity sector row.

Steps	Old columns	New rows	Description	Feature
a	12 Electricity Production Sectors	12 Electricity Production Sectors	Estimate the house-service consumption of power plants	3
b	Final Use	T&D	Determine the final use of electricity	5
c	T&D	T&D	Estimate the line loss and obtain the total amount of electricity transactions	4
d	Non-electricity Sectors with CPPs	Six kinds of Coal-fired Units	Describe the electricity from CPPs	2
e	All Non-electricity Sectors	T&D	Determine the use of electricity from the grid	1, 2
f	T&D	12 Electricity Production Sectors	Estimate electricity transactions	4

The ‘old row’ (i.e. the row of the electricity sector) and the ‘new columns’ (the same as the old columns) are not shown.

## Data Records

The CEFIO dataset and supporting documents are published in Figshare^37^. There are seven items in the repository.

- **2018 CEFIO Table with 166-commodity by 166-commodity:** The most disaggregated table with 166 sectors. The table contains three parts: (1) the first quadrant at the upper left, which comprises the intermediate flows among 166 sectors; (2) the second quadrant at the upper right, which refers to gross output, imports, and the six final use categories (i.e. rural household consumption expenditure, urban household consumption expenditure, government consumption expenditure, gross fixed capital formation, changes in inventories, and exports); and (3) the third quadrant at the lower left, which consists of total inputs and value-added (including compensation of employees, net taxes on production, depreciation of fixed assets, and operating surplus). To maintain consistency with the official IOT, all data were calculated at producer prices in 2018 with a monetary unit of 10 thousand CNY. The CEFIO dataset is stored as an Excel file (available in English and Chinese) and a MATLAB Data file.
- **2018 CEFIO Table with 55-commodity by 55-commodity:** A more aggregated version that was used. The table was processed using a 166-sector version. Excel and MATLAB Data files are provided.
- **Bridge Matrix:** The binary matrix that was used to merge 166 sectors into 55 sectors. The matrix is stored in an Excel spreadsheet.
- **Commodity Sector Classification:** The classification that was used by the CEFIO dataset in the 166- and 55-sector versions. It is provided in English and Chinese and saved in an Excel spreadsheet.
- **Codes:** Four MATLAB Code files were used to process data. The file ‘replication’ is the code that can replicate the entire process of CEFIO dataset construction. The file ‘gras’ is a function script that can implement the GRAS balancing algorithm. Because the MRAS is just the RAS with additional information and the GRAS can completely implement the RAS algorithm^39^, we exclusively used the GRAS code for balancing in practice, although the GRAS and the MRAS were distinguished in the text. The file ‘merge’ is used to merge the 166 sectors into 55 sectors. The file ‘comparison’ replicates the calculation of the comparison between the CEFIO dataset and the official IOT.
- **External Data:** Three Excel files were used to generate the CEFIO dataset. The file ‘raw_macro’ stores the macro data we used, including the data from the statistics department and the CEC^30,32–35^. The file ‘raw_micro’ stores the micro data we used, which is obtained from our unit-level cost database^31^. The file ‘adjustment’ stores all of our manual adjustments during CEFIO construction.
- **Discrepancy:** Two MATLAB Data files were generated from the replication code. The two files describe the discrepancy between the results from the PSM and rationality checks, showing the role of manual adjustments.

## Technical Validation

### Comparison with the official IOT

The 2018 CEFIO dataset is based on the official IOT but is prioritised by statistical calibres and raw data. Therefore, we adjusted the original IOT before disaggregation (with the support of an expert from NBS DNA). Guided by statistical calibres, the total interindustry transactions among the 14 new sectors in the CEFIO was 2412.0 billion CNY, which was nearly 30% larger than the intraindustry input of the original sector (1862.0 billion CNY) in the official IOT. This difference is mainly owing to the CEFIO that revealed 2053.8 billion CNY sales by electricity production sectors to T&D, and 132.3 billion CNY for house-service consumption and abandonment, while the official IOT ignores these features without the support of detailed electricity information.

We used the MRAS to rebalance the IOT after adjusting the original sector’s intraindustry input. However, the algorithm affects intermediate flows, particularly in common sectors. Thus, we compared the intermediate flows of CEFIO (denoted by ${z}_{ij}^{CEFIO}$) with those of the official IOT (denoted by ${z}_{ij}^{OIOT}$). We followed existing research^42–44^, selecting three indicators to estimate the matrix similarity. The mean absolute percentage error (MAPE) and the Isard-Romanoff similarity index (DSIM) are relative distance measures. The absolute psi statistic (ABSPSI) is a representative information-theoretic statistic used to assess the similarity between matrices. For all three measures, the smaller the values, the more similar the matrices. The mathematical expressions for these indicators are shown in Eqs. 5–7:

5 $$\begin{array}{c}MAPE=\frac{100}{m\times n}\mathop{\sum }\limits_{i=1}^{m}\mathop{\sum }\limits_{j=1}^{n}\left|\frac{{z}_{ij}^{CEFIO}-{z}_{ij}^{OIOT}}{{z}_{ij}^{OIOT}}\right|\end{array}$$

6 $$\begin{array}{c}DSIM=\frac{1}{m\times n}\mathop{\sum }\limits_{i=1}^{m}\mathop{\sum }\limits_{j=1}^{n}\frac{\left|{z}_{ij}^{CEFIO}-{z}_{ij}^{OIOT}\right|}{\left|{z}_{ij}^{CEFIO}\right|+\left|{z}_{ij}^{OIOT}\right|}\end{array}$$

7 $$\begin{array}{c}ABSPSI=\mathop{\sum }\limits_{i=1}^{m}\mathop{\sum }\limits_{j=1}^{n}{p}_{ij}\left|ln\left(\frac{{p}_{ij}}{{s}_{ij}}\right)\right|+\mathop{\sum }\limits_{i=1}^{m}\mathop{\sum }\limits_{j=1}^{n}{q}_{ij}\left|ln\left(\frac{{q}_{ij}}{{s}_{ij}}\right)\right|\end{array}$$

where,

$${p}_{ij}=\frac{{z}_{ij}^{CEFIO}}{{\sum }_{i=1}^{m}{\sum }_{j=1}^{n}{z}_{ij}^{CEFIO}},{q}_{ij}=\frac{{z}_{ij}^{OIOT}}{{\sum }_{i=1}^{m}{\sum }_{j=1}^{n}{z}_{ij}^{OIOT}},{s}_{ij}=\frac{{p}_{ij}+{q}_{ij}}{2}$$

Two points should be noted. First, the electricity sector was excluded from the comparison because the original and new sectors were not comparable via these indicators because of their varied dimensions. Second, all the measures could not always be calculated directly during the comparison because ${z}_{ij}^{CEFIO}$ and ${z}_{ij}^{OIOT}$ (and thus, *p*_*ij*_ and *q*_*ij*_) may be zero. Fortunately, the MRAS algorithm is a zero-preserving algorithm that ensures ${z}_{ij}^{CEFIO}$ is zero if the corresponding element ${z}_{ij}^{OIOT}$ is zero. Therefore, we could confidently set them to zero when they could not be calculated.

**Fig. 3:**
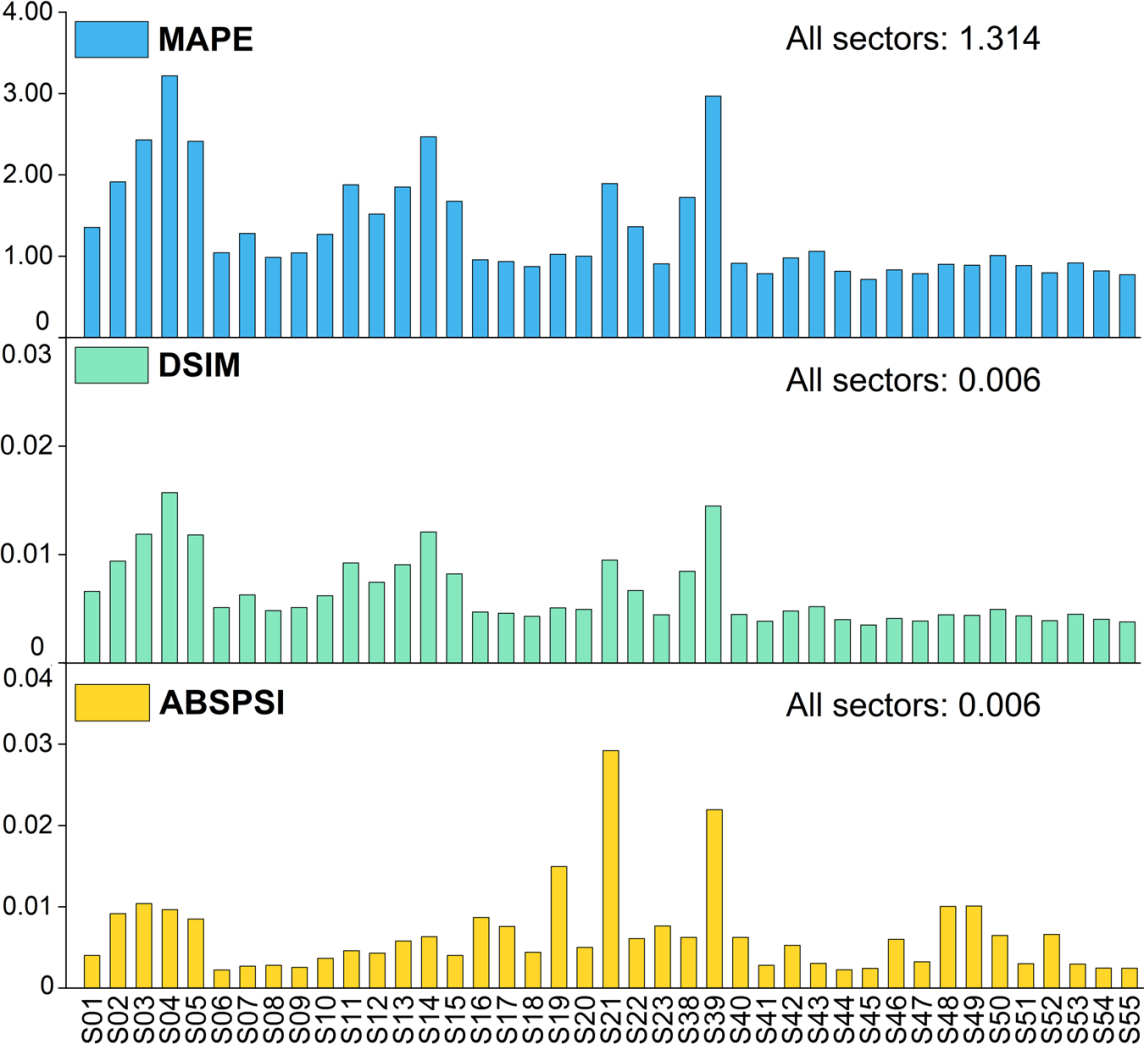
Comparison of intermediate flows of common sectors between the CEFIO and official IOT in aggregated form. All measures are dimensionless. All equations omit the j-summation when computing the sector-specific indicators.

Figure 3 illustrates the results of the three comparison statistics for the pairs of the 2018 CEFIO and official IOT with 41 common sectors in the aggregated versions. In common cases, MAPE values range from 0 to 100. The MAPE estimate for all sectors was 1.314, reflecting the high similarity between the two matrices. The sector-specific maximum MAPE was found in *Mining Products of Metal Ores* (S04) with an MAPE of 3.219, whereas the minimum was 0.715 for *Finance* (S45). The results of the DSIM were similar to those of the MAPE. There were smaller relative differences in the counterparts of all tertiary sectors (S41 to S55), where the DSIM values were below the overall results for all sectors (0.006). The ABSPSI is an information-based indicator with a value of 0.006 for all sectors. Specifically, the largest was 0.029 for *Measuring Instruments* (S21), while the smallest was 0.002 for *Foods and Tobacco* (S06). The similarity estimates for the most disaggregated CEFIO table, including the 152 common sectors identical to those in the official IOT, are shown in Table S2. In summary, our adjustments had as little influence on other intermediate flows as possible.

### Comparisons with relevant studies

**Fig. 4:**
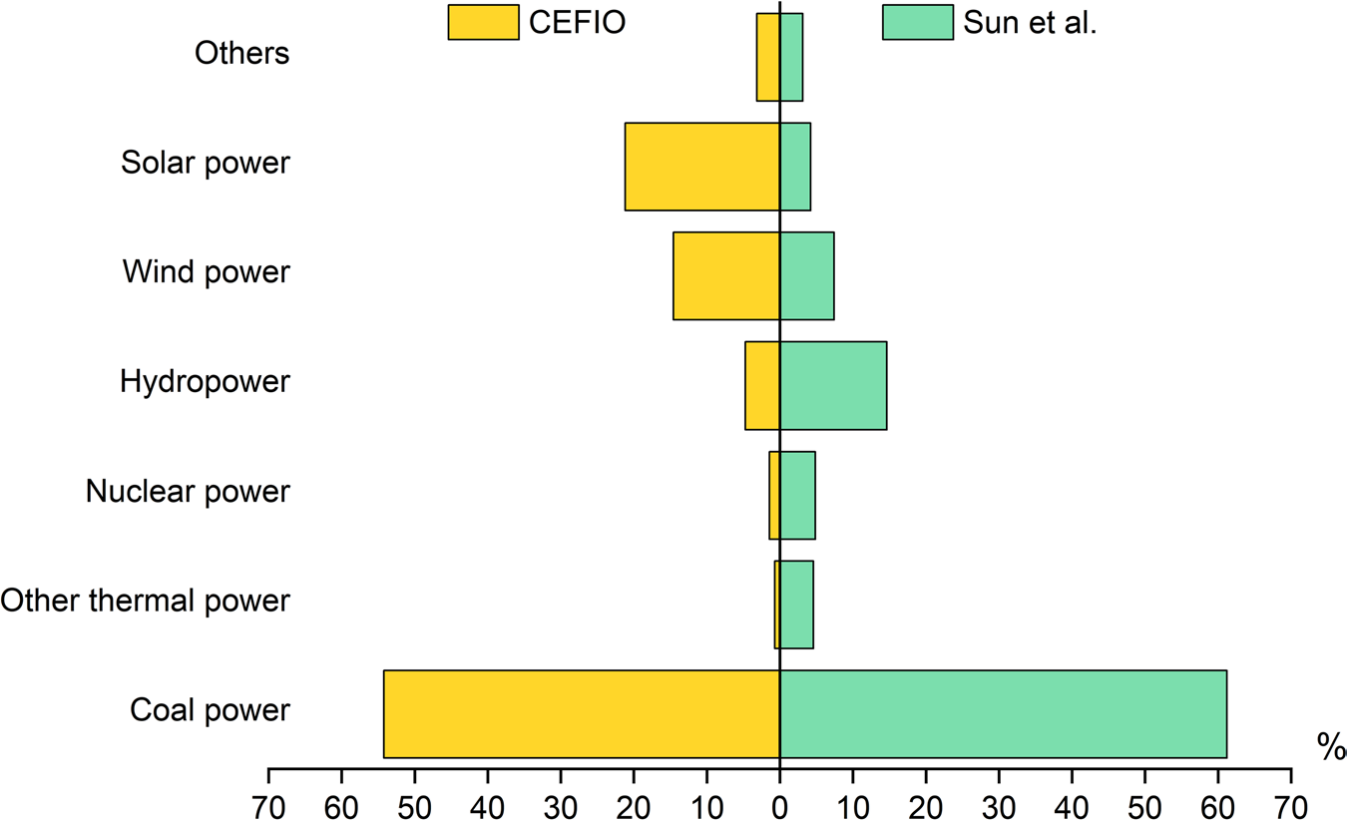
Comparison of the structure of purchases by electricity production sectors from equipment manufacturing. For sectoral consistency, equipment manufacturing in Sun *et al*. was compared with the sector aggregated for 33 sectors (from S067 to S099) in the CEFIO with the 166-sector classification, and gas power in Sun *et al*. was compared with *Other Thermal Power* in the CEFIO. The structure in Sun *et al*. was used to disaggregate the official IOT for 2017 with a one-year distance to the CEFIO. However, this was the only data available.

It should be noted that none of the relevant Chinese IOTs are publicly available, which means that a comparison of elements between input matrices is impossible. Therefore, we alternatively compared the structure of purchases by electricity production sectors from equipment manufacturing between our CEFIO dataset and that of Sun *et al*^16^. (Fig. 4). In the CEFIO, the purchase share of coal power was approximately 7% lower than that in Sun *et al*. (54% versus 61%), which indicates high similarity. However, the shares of the three renewable power sources revealed the differences between the CEFIO and Sun *et al*. We allocated larger proportions to solar power (21%) and wind power (14%), while larger proportions were given to hydropower (14%) in Sun *et al*. This discrepancy mainly arose from the fact that solar and wind generation are generally less stable than hydropower generation and rely heavily on inverters and storage facilities, which belong to *Equipment for Power Transmission and Distribution and Control* (S085).

We also made a general comparison with relevant studies that referred to Wolsky’s approach^8,14,15,28,45^, in which the output share of T&D in the original sector was 10% lower than that of electricity production in China (almost 45% versus 55%). However, this proportion for disaggregation in China was not realistic, because the output (or sales revenue) of T&D ought to be larger than that of electricity production. In the CEFIO dataset, the output share of the *Power Transmission and Distribution* sector was 61.2% according to the Fourth National Economic Census^32^, while the 12 electricity production sectors accounted for 35.3%, and the rest belonged to the *Production and Distribution of Steam*.

The CEFIO dataset considers more detailed features of the electricity structure, such as the share of CPPs for common sectors, LLR for T&D, and the abandonment rate for renewable power, which are not reflected in existing studies. Moreover, rationality checks and coordination in this study were mainly based on our micro database for unit-level coal power^31^, thus ensuring the accuracy of key input flows such as the house-service consumption of the varying installed capacities of coal power. A comparison of features between the CEFIO and relevant studies is shown in Table 6. Consequently, we provide a more rigorous and robust CEFIO dataset with detailed coal power and alternative energy sectors for the year 2018.

**Table 6:** Comparison of electricity features used in the disaggregation between the CEFIO and previous studies.

Categories	Indicators	Kang *et al.*^14^	Lindner *et al.*^28^	Ma *et al.*^45^	CEFIO 2018
**Thermal power**					
Coal power	Gross operations and maintenance cost	✓	✓	✓	✓
	Coal consumption	✓	✓		✓
	Electricity generation	✓	✓		✓
	Electricity sales revenue	✓	✓		✓
	Heat sales revenue	✓			✓
	Coal price	✓			✓
	Feed-in tariff			✓	✓
	House-service consumption				✓
	CPPs share				✓
	Depreciation charge				✓
	Profit before tax				✓
Other thermal power	Electricity generation	✓	✓	✓	✓
	Gas power price				✓
**Renewable power**					
Wind power	Electricity generation	✓	✓	✓	✓
	Sales revenue			✓	✓
	Abandonment rate				✓
Solar power	Electricity generation	✓	✓	✓	✓
	Sales revenue			✓	✓
	Abandonment rate				✓
**T&D**					
T&D	Sales revenue			✓	✓
	LLR				✓

### Supplementary information

Supplementary Information

## Data Availability

All the codes with comments that were used to generate the CEFIO dataset are available in Figshare^37^.
