# Supplementary material for: Chinese electricity-focused input-output dataset with detailed coal power and alternative energy for 2018
Source: Sci Data. 2023 Aug 22;10:553. doi: 10.1038/s41597-023-02466-8 (PMC10444898; doi:10.1038/s41597-023-02466-8)
Supplement: Supplementary file 1 — Supplementary Information [file 41597_2023_2466_MOESM1_ESM.docx]

**Supplementary Information**

| **Label** | **Title** |
| --- | --- |
| Table S1 | Comparison of first-step disaggregation results before and after rationality checking, coordinating, and balancing. |
| Table S2 | Comparison of intermediate flows of the 152 common sectors between the CEFIO and official IOT. |

| **Sector** | **Before rationality checks** | | | | | | | **After rationality checks** | | | | | | |
| --- | --- | --- | --- | --- | --- | --- | --- | --- | --- | --- | --- | --- | --- | --- |
|  | VA001 | VA002 | VA003 | VA004 | VA | TI | VAR (%) | VA001 | VA002 | VA003 | VA004 | VA | TI | VAR (%) |
| Thermal Power | 136 | 151 | 169 | 40 | 496 | 1550 | 32.03 | 139 | 131 | 173 | 41 | 484 | 1550 | 31.20 |
| Hydropower | 43 | 81 | 89 | 72 | 285 | 302 | 94.32 | 44 | 70 | 91 | 73 | 278 | 302 | 91.86 |
| Nuclear Power | 3 | -8 | 25 | 25 | 46 | 98 | 46.99 | 3 | -9 | 26 | 25 | 45 | 98 | 45.76 |
| Wind Power | 10 | -6 | 82 | 50 | 136 | 164 | 83.03 | 10 | -7 | 81 | 49 | 133 | 164 | 80.87 |
| Solar Power | 6 | 6 | 32 | 23 | 67 | 88 | 75.68 | 5 | 5 | 32 | 23 | 65 | 88 | 73.71 |
| Others | 14 | -6 | 14 | 8 | 30 | 72 | 41.40 | 14 | -7 | 14 | 8 | 29 | 72 | 40.32 |
| T&D | 329 | 107 | 433 | 90 | 960 | 3946 | 24.32 | 326 | 91 | 429 | 89 | 935 | 3946 | 23.68 |
| Steam | 58 | **-56** | 22 | -4 | 20 | 228 | **8.56** | 58 | **-4** | 22 | -4 | 72 | 228 | **31.62** |

**Table S1.** Comparison of first-step disaggregation results before and after rationality checking, coordinating, and balancing. ‘VA001’ to ‘VA004’ refer to *Compensation of Employees*, *Net Taxes on Production*, *Depreciation of Fixed Assets*, and *Operating Surplus*, respectively. ‘VA’ stands for value-added. ‘TI’ stands for total inputs. ‘VAR’ stands for the value-added rate. The main changes are highlighted in bold in the table.

| **Sector** | **MAPE** | **DSIM** | **ABSPSI** |
| --- | --- | --- | --- |
| S001 | 1.025 | 0.005 | 0.005 |
| S002 | 0.706 | 0.003 | 0.005 |
| S003 | 0.648 | 0.003 | 0.002 |
| S004 | 0.633 | 0.003 | 0.004 |
| S005 | 0.738 | 0.004 | 0.005 |
| S006 | 1.432 | 0.007 | 0.009 |
| S007 | 1.730 | 0.008 | 0.011 |
| S008 | 2.593 | 0.013 | 0.011 |
| S009 | 2.136 | 0.010 | 0.010 |
| S010 | 2.005 | 0.010 | 0.008 |
| S011 | 0.770 | 0.004 | 0.008 |
| S012 | 0.704 | 0.003 | 0.002 |
| S013 | 0.690 | 0.003 | 0.002 |
| S014 | 0.691 | 0.003 | 0.002 |
| S015 | 0.850 | 0.004 | 0.003 |
| S016 | 0.594 | 0.003 | 0.002 |
| S017 | 0.542 | 0.003 | 0.002 |
| S018 | 0.709 | 0.003 | 0.003 |
| S019 | 0.620 | 0.003 | 0.002 |
| S020 | 0.563 | 0.003 | 0.003 |
| S021 | 0.703 | 0.003 | 0.003 |
| S022 | 0.651 | 0.003 | 0.002 |
| S023 | 0.585 | 0.003 | 0.003 |
| S024 | 0.681 | 0.003 | 0.004 |
| S025 | 0.595 | 0.003 | 0.004 |
| S026 | 0.515 | 0.003 | 0.009 |
| S027 | 0.893 | 0.004 | 0.003 |
| S028 | 0.650 | 0.003 | 0.003 |
| S029 | 0.774 | 0.004 | 0.003 |
| S030 | 0.813 | 0.004 | 0.003 |
| S031 | 0.722 | 0.004 | 0.003 |
| S032 | 0.697 | 0.003 | 0.003 |
| S033 | 0.564 | 0.003 | 0.002 |
| S034 | 0.611 | 0.003 | 0.003 |
| S035 | 0.778 | 0.004 | 0.003 |
| S036 | 0.659 | 0.003 | 0.003 |
| S037 | 1.068 | 0.005 | 0.005 |
| S038 | 0.607 | 0.003 | 0.003 |
| S039 | 0.730 | 0.004 | 0.004 |
| S040 | 0.597 | 0.003 | 0.004 |
| S041 | 1.202 | 0.006 | 0.004 |
| S042 | 1.522 | 0.007 | 0.008 |
| S043 | 2.359 | 0.012 | 0.006 |
| S044 | 1.544 | 0.008 | 0.007 |
| S045 | 1.141 | 0.006 | 0.006 |
| S046 | 0.850 | 0.004 | 0.005 |
| S047 | 1.082 | 0.005 | 0.005 |
| S048 | 0.982 | 0.005 | 0.005 |
| S049 | 0.671 | 0.003 | 0.004 |
| S050 | 0.723 | 0.004 | 0.004 |
| S051 | 1.158 | 0.006 | 0.004 |
| S052 | 0.756 | 0.004 | 0.004 |
| S053 | 0.818 | 0.004 | 0.003 |
| S054 | 2.080 | 0.010 | 0.008 |
| S055 | 0.989 | 0.005 | 0.005 |
| S056 | 1.303 | 0.006 | 0.006 |
| S057 | 1.010 | 0.005 | 0.006 |
| S058 | 1.120 | 0.006 | 0.006 |
| S059 | 1.421 | 0.007 | 0.007 |
| S060 | 1.385 | 0.007 | 0.007 |
| S061 | 1.860 | 0.009 | 0.008 |
| S062 | 1.868 | 0.009 | 0.010 |
| S063 | 2.399 | 0.012 | 0.008 |
| S064 | 2.287 | 0.011 | 0.009 |
| S065 | 1.091 | 0.005 | 0.005 |
| S066 | 1.231 | 0.006 | 0.004 |
| S067 | 0.654 | 0.003 | 0.012 |
| S068 | 0.537 | 0.003 | 0.014 |
| S069 | 0.596 | 0.003 | 0.011 |
| S070 | 0.551 | 0.003 | 0.006 |
| S071 | 0.533 | 0.003 | 0.013 |
| S072 | 0.589 | 0.003 | 0.008 |
| S073 | 0.587 | 0.003 | 0.010 |
| S074 | 0.519 | 0.003 | 0.010 |
| S075 | 0.550 | 0.003 | 0.009 |
| S076 | 0.534 | 0.003 | 0.006 |
| S077 | 0.566 | 0.003 | 0.011 |
| S078 | 0.530 | 0.003 | 0.007 |
| S079 | 0.499 | 0.002 | 0.004 |
| S080 | 0.539 | 0.003 | 0.004 |
| S081 | 0.633 | 0.003 | 0.014 |
| S082 | 0.862 | 0.004 | 0.018 |
| S083 | 0.540 | 0.003 | 0.008 |
| S084 | 0.718 | 0.004 | 0.014 |
| S085 | 1.795 | 0.009 | 0.039 |
| S086 | 0.584 | 0.003 | 0.005 |
| S087 | 0.700 | 0.003 | 0.006 |
| S088 | 0.521 | 0.003 | 0.008 |
| S089 | 0.527 | 0.003 | 0.013 |
| S090 | 0.628 | 0.003 | 0.006 |
| S091 | 0.503 | 0.002 | 0.004 |
| S092 | 0.984 | 0.005 | 0.020 |
| S093 | 0.563 | 0.003 | 0.005 |
| S094 | 0.488 | 0.002 | 0.005 |
| S095 | 0.594 | 0.003 | 0.008 |
| S096 | 1.489 | 0.007 | 0.030 |
| S097 | 0.722 | 0.004 | 0.006 |
| S098 | 1.342 | 0.007 | 0.009 |
| S099 | 0.549 | 0.003 | 0.008 |
| S114 | 1.165 | 0.006 | 0.006 |
| S115 | 1.991 | 0.010 | 0.022 |
| S116 | 0.533 | 0.003 | 0.006 |
| S117 | 0.541 | 0.003 | 0.005 |
| S118 | 0.542 | 0.003 | 0.005 |
| S119 | 0.539 | 0.003 | 0.005 |
| S120 | 0.500 | 0.002 | 0.014 |
| S121 | 0.530 | 0.003 | 0.011 |
| S122 | 0.377 | 0.002 | 0.003 |
| S123 | 0.552 | 0.003 | 0.003 |
| S124 | 1.510 | 0.007 | 0.006 |
| S125 | 1.427 | 0.007 | 0.007 |
| S126 | 0.687 | 0.003 | 0.004 |
| S127 | 0.465 | 0.002 | 0.006 |
| S128 | 0.490 | 0.002 | 0.005 |
| S129 | 0.542 | 0.003 | 0.005 |
| S130 | 0.416 | 0.002 | 0.004 |
| S131 | 0.457 | 0.002 | 0.005 |
| S132 | 1.602 | 0.008 | 0.012 |
| S133 | 0.377 | 0.002 | 0.003 |
| S134 | 0.504 | 0.002 | 0.004 |
| S135 | 0.343 | 0.002 | 0.002 |
| S136 | 0.816 | 0.004 | 0.004 |
| S137 | 0.509 | 0.003 | 0.003 |
| S138 | 0.589 | 0.003 | 0.003 |
| S139 | 0.489 | 0.002 | 0.007 |
| S140 | 0.355 | 0.002 | 0.002 |
| S141 | 0.378 | 0.002 | 0.002 |
| S142 | 0.383 | 0.002 | 0.002 |
| S143 | 0.408 | 0.002 | 0.003 |
| S144 | 0.248 | 0.001 | 0.003 |
| S145 | 0.371 | 0.002 | 0.004 |
| S146 | 0.526 | 0.003 | 0.006 |
| S147 | 0.381 | 0.002 | 0.006 |
| S148 | 0.347 | 0.002 | 0.003 |
| S149 | 0.648 | 0.003 | 0.011 |
| S150 | 0.606 | 0.003 | 0.012 |
| S151 | 0.427 | 0.002 | 0.006 |
| S152 | 0.410 | 0.002 | 0.019 |
| S153 | 0.633 | 0.003 | 0.008 |
| S154 | 0.704 | 0.003 | 0.005 |
| S155 | 0.655 | 0.003 | 0.003 |
| S156 | 0.484 | 0.002 | 0.004 |
| S157 | 0.519 | 0.003 | 0.007 |
| S158 | 0.573 | 0.003 | 0.003 |
| S159 | 0.694 | 0.003 | 0.005 |
| S160 | 0.516 | 0.003 | 0.003 |
| S161 | 0.439 | 0.002 | 0.003 |
| S162 | 0.497 | 0.002 | 0.004 |
| S163 | 0.550 | 0.003 | 0.003 |
| S164 | 0.473 | 0.002 | 0.004 |
| S165 | 0.271 | 0.001 | 0.005 |
| S166 | 0.351 | 0.002 | 0.003 |
| All sectors | 0.809 | 0.004 | 0.007 |

**Table S2.** Comparison of intermediate flows of the 152 common sectors between the CEFIO and official IOT.
